# SNX31: A Novel Sorting Nexin Associated with the Uroplakin-Degrading Multivesicular Bodies in Terminally Differentiated Urothelial Cells

**DOI:** 10.1371/journal.pone.0099644

**Published:** 2014-06-10

**Authors:** Neide Vieira, Fang-Ming Deng, Feng-Xia Liang, Yi Liao, Jennifer Chang, Ge Zhou, Weiyue Zheng, Jean-Pierre Simon, Mingxiao Ding, Xue-Ru Wu, Rok Romih, Gert Kreibich, Tung-Tien Sun

**Affiliations:** 1 Department of Cell Biology, NYU Cancer Institute, NYU Langone Medical Center, New York, New York, United States of America; 2 Department of Pathology, NYU Cancer Institute, NYU Langone Medical Center, New York, New York, United States of America; 3 Department of Dermatology, NYU Cancer Institute, NYU Langone Medical Center, New York, New York, United States of America; 4 Department of Biochemistry and Molecular Pharmacology, NYU Cancer Institute, NYU Langone Medical Center, New York, New York, United States of America; 5 College of Life Sciences, Peking University, Beijing, People's Republic of China; 6 Department of Urology; NYU Cancer Institute, NYU Langone Medical Center, New York University, New York, New York, United States of America; 7 Veterans Affairs New York Harbor Healthcare Systems, Manhattan Campus, New York, New York, United States of America; 8 Institute of Cell Biology, Faculty of Medicine, University of Ljubljana, Ljubljana, Slovenia; Dalhousie University, Canada

## Abstract

Uroplakins (UP), a group of integral membrane proteins, are major urothelial differentiation products that form 2D crystals of 16-nm particles (urothelial plaques) covering the apical surface of mammalian bladder urothelium. They contribute to the urothelial barrier function and, one of them, UPIa, serves as the receptor for uropathogenic Escherichia coli. It is therefore important to understand the mechanism by which these surface-associated uroplakins are degraded. While it is known that endocytosed uroplakin plaques are targeted to and line the multivesicular bodies (MVBs), it is unclear how these rigid-looking plaques can go to the highly curved membranes of intraluminal vesicles (ILVs). From a cDNA subtraction library, we identified a highly urothelium-specific sorting nexin, SNX31. SNX31 is expressed, like uroplakins, in terminally differentiated urothelial umbrella cells where it is predominantly associated with MVBs. Apical membrane proteins including uroplakins that are surface biotin-tagged are endocytosed and targeted to the SNX31-positive MVBs. EM localization demonstrated that SNX31 and uroplakins are both associated not only with the limiting membranes of MVBs containing uroplakin plaques, but also with ILVs. SNX31 can bind, on one hand, the PtdIns3P-enriched lipids via its N-terminal PX-domain, and, on the other hand, it binds uroplakins as demonstrated by co-immunoprecipitation and proximity ligation assay, and by its reduced membrane association in uroplakin II-deficient urothelium. The fact that in urothelial umbrella cells MVBs are the only major intracellular organelles enriched in both PtdIns3P and uroplakins may explain SNX31's MVB-specificity in these cells. However, in MDCK and other cultured cells transfected SNX31 can bind to early endosomes possibly via lipids. These data support a model in which SNX31 mediates the endocytic degradation of uroplakins by disassembling/collapsing the MVB-associated uroplakin plaques, thus enabling the uroplakin-containing (but ‘softened’) membranes to bud and form the ILVs for lysosomal degradation and/or exosome formation.

## Introduction

Mammalian bladder epithelium is a stratified squamous epithelium consisting of basal, intermediate and terminally differentiated umbrella cell layers. The umbrella cells are highly flattened (70–100 um in diameter; hence the term ‘umbrella’ cells). They can withstand repeated and extensive stretch during the micturition cycle while maintaining a highly effective permeability barrier [1]–[5]. Perhaps related to such specialized functions, the apical surface of the umbrella cell is covered by 2D crystals (‘urothelial plaques’) of hexagonally packed 16-nm particles consisting of four major integral membrane proteins, i.e., uroplakin Ia (UPIa, 27-kDa), UPIb (28-kDa), UPII (15-kDa) and UPIIIa (47-kDa) [5]–[8]. Uroplakins are functionally important, because knockout of UPII and IIIa genes compromises the urothelial barrier function [9]–[12]. Moreover, one of the uroplakins, UPIa, can serve as the urothelial receptor for the type 1-fimbriated E. coli that causes a great majority of urinary tract infections [13]–[17].

Of the four major uroplakins, two, UPIa and UPIb, are tetraspanins (40% identity) [18], while UPII and UPIIIa share a stretch of largely luminal ∼12 amino acid residues near their single transmembrane domain [19], [20]. The four uroplakins initially form two heterodimers (UPIa/II and Ib/IIIa), which acquire the ability to exit the ER [21]–[24]. Two dimers then form a heterotetramer (a ‘subunit’), six of which form a 16-nm particle [24]–[26] that are delivered into small discoidal vesicles. As the 2D crystals of uroplakins progressively enlarge, the vesicles become flattened to eventually consist of only two large uroplakin plaques joined by a hinge area (fusiform vesicles; [27], [28]). These mature uroplakin-delivering vesicles can then fuse with the apical surface [5], [24], [28], [29]. Of the four uroplakins, UPIIIa has the longest cytoplasmic domain of about 50 amino acid residues that may mediate membrane:cytoskeletal interaction and signal transduction [5], [20], [30].

Given the functional importance of the apical surface-associated uroplakins, their endocytic retrieval is likely to be tightly regulated [31]–[33]. It has been a central dogma of the urothelial biology field that the uroplakin-delivering fusiform vesicles can be induced by bladder distension to fuse with the apical surface, and that the surface-associated plaques can later be retrieved, upon bladder contraction, to re-form cytoplasmic fusiform vesicles thus achieving reversible adjustment of the apical cell surface area [2], [3], [33], [34]. This view is not supported, however, by tracer studies indicating that internalized luminal plaques mainly become associated with the multivesicular bodies followed by lysosomal degradation [35], [36]. A critical and as yet unanswered question about the endocytic degradation of uroplakins is: how can the rigid-looking uroplakin plaques, once become incorporated into the limiting membranes of the MVBs, bud into the lumen of MVB to form the small intraluminal vesicles, that have an extremely high curvature, for lysosomal degradation [37]?

To identify urothelium-specific genes, we previously generated a urothelium-specific cDNA library by suppression subtractive hybridization to eliminate cDNAs that are also present in ten other non-urothelial tissues including kidney, lung, spleen, skeletal muscle, esophagus, stomach, intestine, colon, brain, and liver [38]. This subtraction library achieved ∼1,000-fold enrichment of urothelial-specific cDNA, as evidenced by a >10-fold increase in the cDNA of uroplakin Ib, a urothelial marker, and a >100-fold reduction in actin cDNA [38]. From this cDNA library, we originally identified several secretory proteins including tissue-type plasminogen activator, urokinase and a serine protease inhibitor PP5, that are made as major urothelial differentiation products that are secreted into the urine, thus establishing that bovine urothelium can function not only as a permeability barrier but also as a source of some urinary proteins [38].

Here we report the identification, from this urothelium-enriched cDNA library, of a novel sorting nexin, SNX31, that is co-expressed with uroplakins in terminally differentiated urothelial umbrella cells. The sorting nexins family proteins are involved in regulating protein trafficking, and their defects have been linked to several diseases including Alzheimer's and several infections [39], [40]. SNX31 binds the uroplakin- as well as PtdIns3P-enriched membranes of the multivesicular bodies (and their intraluminal vesicles) that are the most prominent endosomal compartment in urothelial umbrella cells. Biotinylated, surface-associated uroplakins are endocytosed into the SNX31-positive mutlivesicular vesicles (MVBs), where uroplakins and SNX31 co-localize not only at the limiting plaques, but also at the highly curved membranes of the small intraluminal vesicles. We propose a model in which the binding of SNX31 to the cytoplasmic domain of uroplakins may cause the dissociation and collapse of the uroplakin particles located at the periphery of the MVB-associated plaques, thus facilitating the budding of the uroplakin-containing membranes to form intraluminal vesicles for lysosomal degradation and/or exosome formation.

## Results

### SNX31 is co-expressed with uroplakins in terminally differentiated urothelial umbrella cells

**Figure 1:**
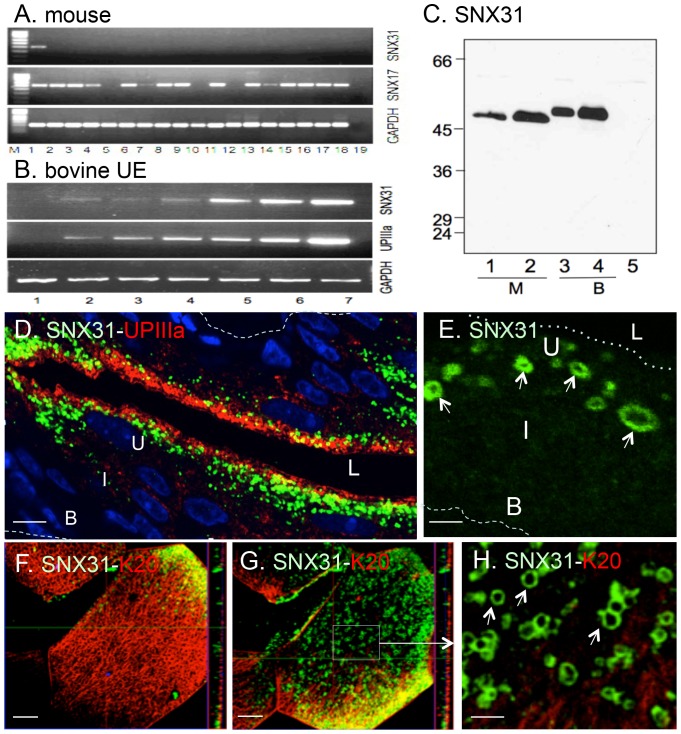
SNX31 is expressed in a urothelium-enriched and differentiation-dependent manner. (**A**) RT-PCR: cDNA's from various mouse tissues were used to amplify sorting nexin 31 (SNX31), SNX17 and glyceraldehyde phosphate dehydrogenase (GAPDH; loading control). Lanes: (1) bladder, (2) kidney medulla, (3) kidney cortex, (4) cornea, (5) trachea, (6) lung, (7) esophagus, (8) spleen, (9) intestine, (10) skeletal muscle, (11) heart, (12) fat (skin), (13) fat (abdomen), (14) vagina, (15) uterus, (16) testis, (17) seminal vesicle, (18) ovary, and (19) water (negative control). M: size markers. (**B**) Differentiation-dependent expression of SNX31 and UPIIIa (assayed by semi-quantitative RT-PCR) in cultured bovine urothelial cells. Lanes: (1) mouse 3T3 cells (negative control), (2) cultured bovine bladder epithelial cells at 50% confluence, (3) 100% confluence, (4) 3 days post confluence, (5) 6 days post-confluence, (6) 9 days post-confluence, and (7) *in vivo* bovine bladder epithelium. (**C**) **Monospecific antibodies to SNX31**. A rabbit antiserum to SNX31 was raised against a synthetic peptide (positions 423–439 in the C-terminal region of mouse SNX31), affinity-purified and used to immunoblot total cellular lysates of: (Lane 1) mouse (M) bladder urothelium, (2) 293T cells transfected with a mouse SNX31 cDNA, (3) bovine (B) bladder urothelium, (4) 293T cells transfected with a bovine SNX31 cDNA, and (5) 293T cells transfected with an empty pcDNA3 plasmid (negative control). Note that the antibody recognized a single band of 51-kDa mouse SNX31 and a 53-kDa bovine SNX31. (**D-E**) **Localization of SNX31 in the mouse urothelium**. A vertical section of mouse bladder was double-stained using a monospecific, rabbit antiserum against SNX31 (green) and a mouse monoclonal antibody against uroplakin IIIa (red). (**E**) A higher magnification view showing the SNX31-positive vesicles. Note in **D** and **E**, that SNX31 was expressed mainly in the terminally differentiated umbrella cells, and that SNX31 vesicles were located slightly below the uroplakin-enriched zone. **(F-H) Localization of SNX31 in a whole-mount, horizontal section of mouse urothelium by double-staining**. Horizontal sections beneath the apical surface of mouse umbrella cells were double-stained for keratin K20 (red) and SNX31 (green). (**F**) Note that K20 forms a subapical, chicken wire-like network with no SNX31 vesicles (except the upper right corner showing the deeper cytoplasm). (**G**) A deeper section (below the K20 positive zone) showing the SNX31-positive vesicles. (**H**) Enlargement of the boxed area in panel G showing intense staining of the periphery of the SNX31-positive vesicles. B (basal cells), I (intermediate cells), U (umbrella cells), dashed line (basement membrane) and dotted line (urothelial apical surface). Note in (D), and in (F) vs. (G), that the SNX31-positive vesicles are located below the subapical Keratin 20 network. Scale bars  = 10 µm (panels D, F and G) or 1 µm (E and H).

From a previously established urothelium-enriched cDNA library [38], we identified a cDNA clone that encoded a sorting nexin that was highly enriched in mouse (Fig. 1A) and bovine bladder (Fig. S1). This cDNA encoded sorting nexin 31 (SNX31) whose expression, like that of uroplakin IIIa, was greatly up-regulated when cultured bovine urothelial cells reached confluence and became more stratified and differentiated (Fig. 1B; [41]), indicating a differentiation-dependent expression.

We generated several affinity-purified rabbit (peptide) antibodies against mouse and bovine SNX31. When used to immunoblot the total mouse and bovine urothelial proteins resolved by SDS-PAGE, these antibodies recognized a single band of SNX31 (51-kDa or 53-kDa for mouse or bovine, respectively; Fig. 1C, lanes 1 and 3); this band was specific as it was undetectable by preimmune antibodies. Immunofluorescent staining of mouse urothelial (vertical) sections using these antibodies revealed that SNX31 was associated with cytoplasmic vesicles in terminally differentiated, superficial umbrella cells (Fig. 1D and E). Double-staining showed that the SNX31-positive vesicles were located lower than uroplakins in the cytoplasm of the umbrella cells (Fig. 1D). Staining of horizontal sections of urothelium in a whole-mount mouse bladder preparation, which offered a better spatial resolution, showed that keratin K20 formed a subapical, chicken wire-like network (Fig. 1F; [42]), and that SNX31-positive vesicles were below this subapical K20-network and the uroplakin-enriched upper cytoplasm (Fig. 1F–H).

### SNX31 is associated with multivesicular bodies in terminally differentiated urothelial umbrella cells

**Figure 2:**
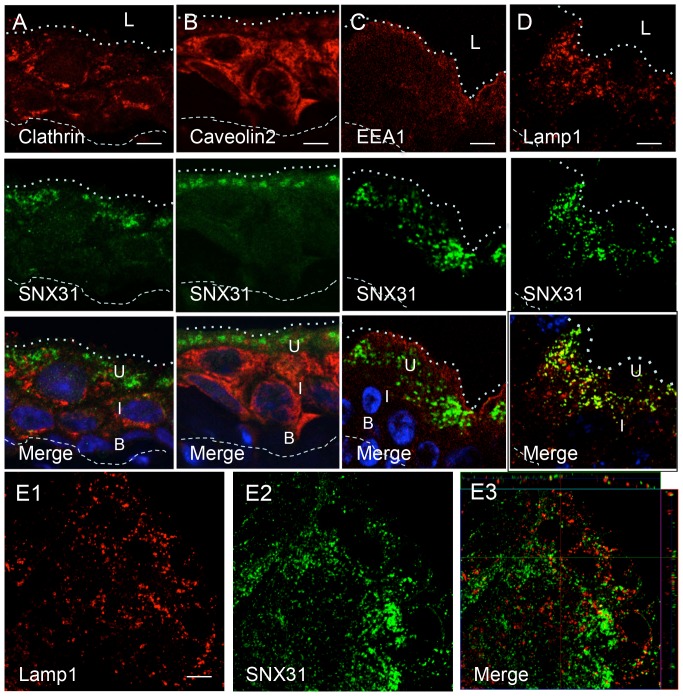
SNX31 is distinguishable from other endocytic marker in mouse urothelial umbrella cells. Vertical sections of mouse urothelium were double-stained for SNX31 (green; middle row) and several known endosomal markers (red; top row): (**A**) Clathrin, (**B**) Caveolin-2, (**C**) EEA1, and (**D**) Lamp1. Merged images with nuclear staining (DAPI) are presented (bottom row). (**E**) A horizontal section from a whole-mounted mouse bladder urothelium double-stained for Lamp1 (E1, red) and SNX31 (E2, green); merged image (E3). Note in (E) the clear separation of SNX31 and Lamp1 staining. Symbols are the same as in Fig. 1. Scale bar  = 10 µm.

**Figure 3:**
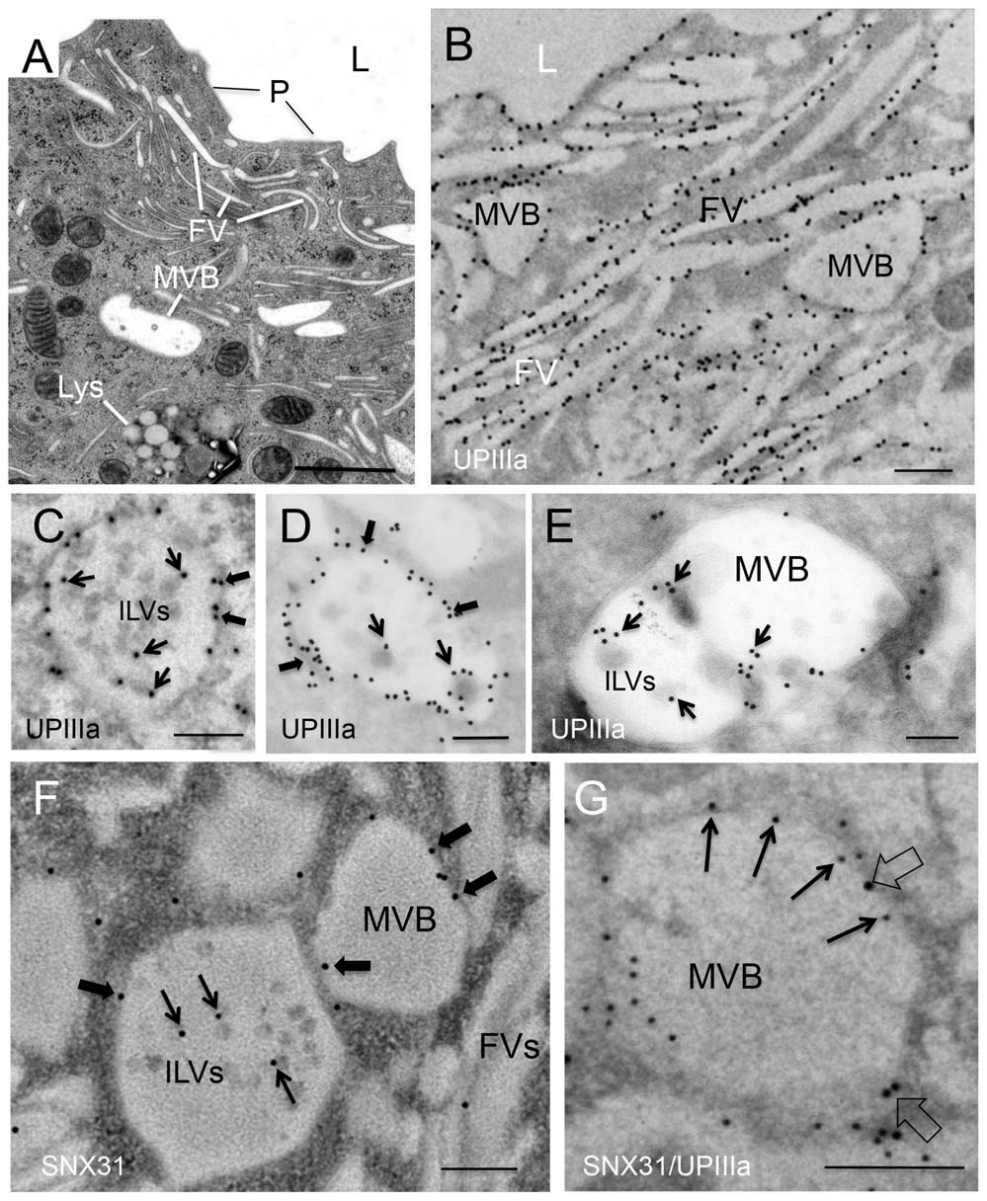
SNX31 co-localizes with uroplakins in the multivesicular bodies of mouse umbrella cells. (**A**) TEM of normal mouse umbrella cells showing apical plaques (P), fusiform vesicles (FV), multivesicular vesicles (MVB) and lysosomes (Lys). (**B–E**) EM localization of uroplakin IIIa using a monoclonal antibody AU1 (B and C: Lowicryl procedure; D and E: cryoEM). Note in (B) the uroplakin staining of apical plaques, fusiform vesicles, and multivesicular bodies (MVBs), in (C) and D) the presence of uroplakin not only at the limiting membranes (thick arrows), but also the intra-luminal vesicles (thin arrows), and in (E) the uroplakin association with some MVB invaginations. (**F**) EM localization of SNX31. Note the detection of SNX31 in both the limiting membranes (thick arrows) and the intra-luminal vesicles (thin arrows), but not in the neighboring fusiform vesicles. (**G**) **Double-labeling of UPIIIa** (10-nm immunogold particles; open arrows) **and SNX31** (5-nm; filled arrows). Note their co-localization at the limiting membranes of MVB. Scale bar  = 1 µm (in panel A), or 0.2 µm (all others).

**Figure 4:**
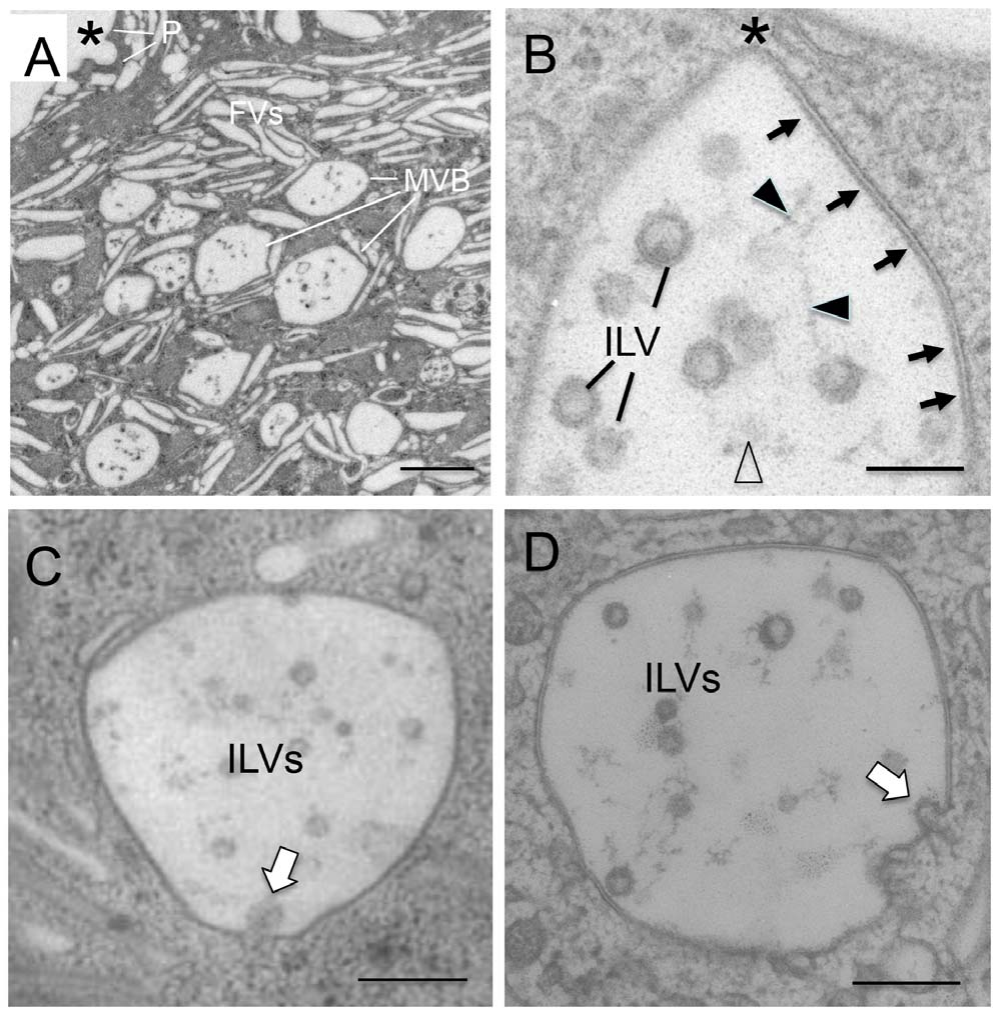
Formation of the intraluminal vesicles via the invagination of the MVB membranes. Normal mouse bladder was fixed by high pressure freezing and freeze-substitution ([29]), cut into 60-nm sections and its urothelial umbrella cells examined by transmission EM. (**A**) An umbrella cell containing many MVBs lined with uroplakin plaques. (**B**) The limiting membranes of MVB consist of uroplakin plaques with thickened, rigid-looking leaflets (arrows) interrupted by a hinge area (asterisk), and the intraluminal vesicles (ILVs) are connected with thin, irregular filaments (filled arrowheads), and amorphous materials (open arrowhead). (**C and D**) Formation of ILVs via the invagination of the limiting membrane of MVBs (open arrows). Scale bar  = 1 µm (A), 0.1 µm (B), or 0.2 µm (C and D).

The SNX31 staining pattern in the umbrella cells was distinct from those of endocytic markers clathrin (Fig. 2A), caveolin 2 (Fig. 2B), EEA1 (Fig. 2C), Rab5a and GTPase Rho A, and the exocytic marker Rab11a (data not shown). Although in vertical sections of mouse urothelium the SNX31-vesicles seemed to be partially overlapping with the late endosome marker Lamp1 (Fig. 2D), high-resolution whole mount microscopy clearly distinguished the SNX31- and Lamp1-positive vesicles (Fig. 2E). Electron microscopy identified these SNX31-positive vesicles as multivesicular bodies (MVBs), which were lined with mature-looking uroplakin plaques and contained many small intraluminal vesicles (ILVs; Figs. 3 and 4).

**Figure 5:**
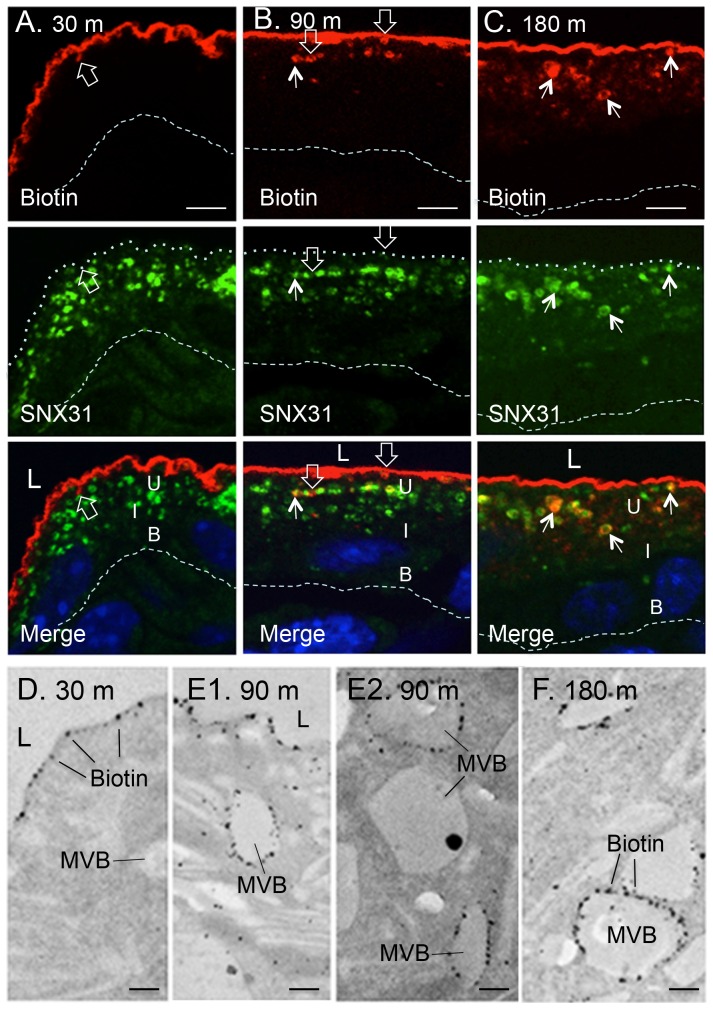
The endocytosed apical urothelial proteins are targeted to the SNX31-positive MVBs. (**A–C**) The luminal surface of the mouse bladder was biotinylated for 15 minutes, followed by a chase period as indicated (30, 90 and 180 min). Samples were co-stained for biotin (red; top row) and SNX31 (green, middle), with merged images shown at the bottom. Note that the internalized, biotinylated proteins (mainly uroplakins; [45]) were initially associated with SNX31-free vesicles (open arrows), and later became co-localized with SNX31 (arrows). (**D–F**) EM immunolocalization showing the progressive association of the endocytosed (biotinylated) surface-proteins (mainly uroplakins) with the multivesicular vesicles (MVBs). Symbols are the same as in Fig. 1. Scale bars  = 10 µm (A to C), or 0.2 µm (D to F).

EM localization showed that uroplakins were associated with the apical surface, fusiform vesicles, the MVB-associated uroplakin plaques as well as ILVs (Fig. 3B–D; [5], [20], [21], [43], [44]). In contrast, SNX31 was associated with only the MVB-associated plaques as well as the ILVs, where they co-localized with uroplakins (Fig. 3F and G).

In a few cases, we found that uroplakin IIIa was localized on membrane budding from the limiting membranes of the MVBs into the lumen (Fig. 3E). To better understand this process, we examined the ultrastructure of urothelial MVBs using high pressure freezing and freeze-substitution which offers a superior resolution over conventional TEM (Fig. 4). Our data indicate that the MVBs are lined with urothelial plaques morphologically indistinguishable from the apical plaques interconnected by hinge areas (Fig. 4A and B), and that ILVs were formed by invagination of the MVB membrane in hinge areas (Fig. 4C and D; open arrows). The fact that such images were quite rare suggests that the invagination process was either rapid, or rare, or both.

To see whether the apical membrane proteins can be delivered to MVBs, we biotinylated the apical surface of mouse bladder urothelium *in situ* using a membrane-impermeable biotinylation reagent, sulfo-N-hydroxysuccinimide [45]. The internalized, biotin-tagged surface proteins, that we showed earlier are mostly uroplakins [45], initially form SNX31-negative vesicles (Fig. 5A and B; open arrows), and later became co-localized with the SNX31-positive MVBs (Fig. 5B and C). Electron microscopy showed that the internalized apical proteins are largely associated with the limiting plaques of the MVBs (Fig. 5D–F). These results indicate that the SNX31-positive MVB plaques are accessible from the apical surface.

### SNX31 interacts with uroplakins

**Figure 6:**
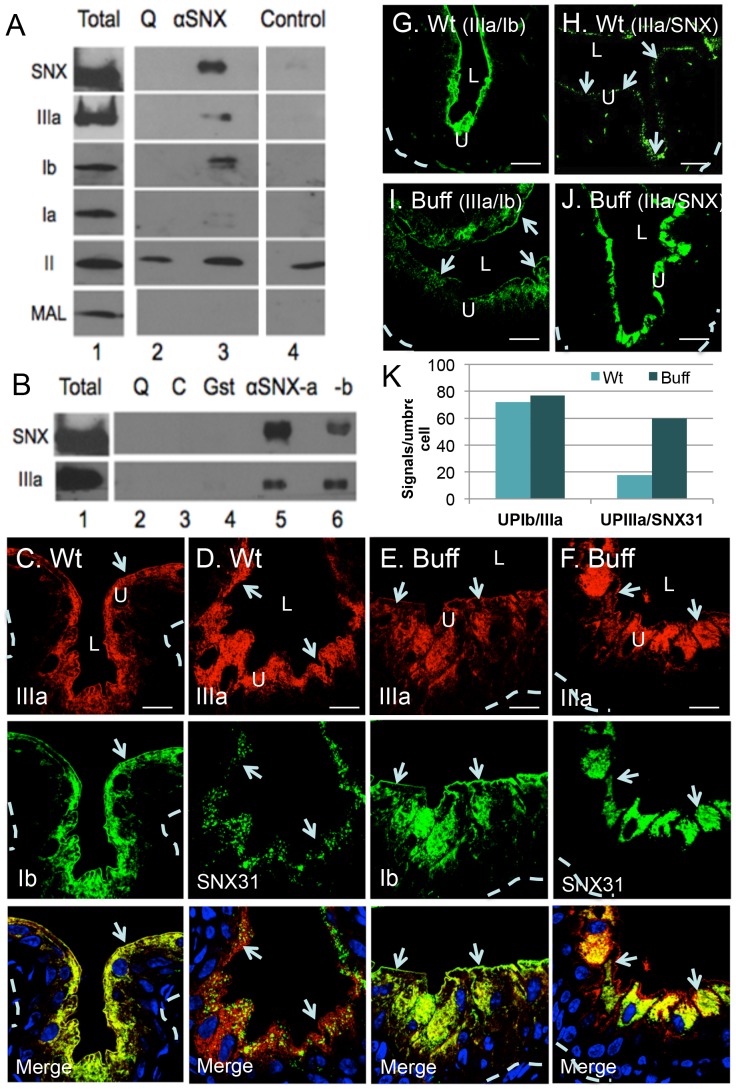
SNX31:uroplakin interactions. (A–B) Co-immunoprecipitation of SNX31 and uroplakins. (**A**): Affinity-purified, monospecific SNX31 antibodies were crosslinked to an activated aminolink-plus resin (see Methods). 300 µg of total mouse urothelial proteins were incubated with the beads for 4h at 4°C, the immunoprecipitated proteins were resolved by SDS-PAGE and detected using antibodies as denoted (all monospecific recognizing a single band that is shown). Beads whose activated crosslinking side chains were quenched (Q), or control beads (C), were used as negative controls. Note that the anti-SNX31 pulled down not only SNX31, but also UPIIIa and UPIb (of the UPIIIa/Ib pair). UPII was pulled down, but the negative controls indicated that this was nonspecific. (**B**) A separate co-immunoprecipitation assay with anti-SNX31 crosslinked to the beads under two different crosslinking conditions (aSNX31a and aSNX31b); anti-Gst beads (Gst) were used as an additional negative control. (**C**) Co-localization of UPIIIa (red) and UPIb (green) in normal mouse urothelium. Note that UPIb and IIIa (UPIb/IIIa pair) co-localized precisely, as expected. (**D**) Co-localization of UPIIIa (red) and SNX31 (green) in normal mouse urothelium. Note the distinct vesicular pattern of SNX31 in umbrella cells. (**E**) Co-localization of UPIIIa (red) and UPIb (green) in the urothelium of the Vps33a mutant (Buff) mouse. (**F**) Co-localization of UPIIIa (red) and SNX31 (green) in the Buff mouse urothelium. Note the tremendously increased amount of SNX31-positive vesicles in (E), comparing with (D). (**G–J**) *In situ* Proximity Ligation Assay (PLA) in mouse umbrella cells. Paraffin sections of the mouse bladder were blocked for 1 h in 1% Fish Gelatin. Antibodies for UPIb/UPIIIa or UPIIIa/SNX31 were used to detect these two proteins. The primary antibodies were also used alone, or omitted, as negative controls (data not shown). PLA signal is shown in green. (**G**) PLA assay of UPIb and UPIIIa interaction in the wild-type mouse urothelium, as a positive control. (**H**) PLA signal of UPIIIa and SNX31 interaction in normal mouse urothelium. Note that although the interaction was less prominent as compared with UPIb/UPIIIa, punctate signals were detected corresponding most likely to MVBs. (**I**) PLA signal of UPIb and UPIIIa interaction in the Buff (Vps33a mutant) mouse urothelium. (**J**) PLA signal of UPIIIa/SNX31 interaction in the Buff mouse urothelium. Note the tremendously increased PLA signals between UPIIIa and SNX31. (**K**) Quantification of the PLA signals using the Duolink Image Tool software: The average numbers of signals per umbrella cells are presented. Arrows mark the apical surface; other symbols are the same as in Fig. 1. Scale bars  = 10 µm (panels C to J)

The above data indicate that SNX31 and uroplakins co-existed in not only the peripheral membranes, but also the ILVs of the MVBs. To determine whether SNX31 directly binds uroplakins, we performed co-immunoprecipitation experiments. Total proteins of mouse urothelial cells were solubilized in a detergent-containing buffer, and immunoprecipitated using a monospecific antibody to SNX31 (Fig. 1C). Immunoblotting of the immunoprecipitated proteins resolved by SDS-PAGE showed the expected 51-kDa SNX31 (Fig. 6A and B). Additional immunoblotting of the same fraction using antibodies to individual uroplakins detected UPIIIa and Ib (of the UPIIIa/Ib pair; Fig. 6A and B); which were not detected in proteins pulled down by the control (quenched) beads. UPIa was not detected and the results of UPII were inconclusive due to nonspecific UPII binding even to the control beads (Fig. 6A and B). Similar results were obtained in three independent experiments (Fig. 6A and B, and data not shown).

To determine the *in situ* subcellular sites of SNX31:uroplakin interaction, we performed proximity ligation assay (PLA; see Methods), which can localize and quantify protein-protein interactions at the single molecule level using standard fluorescent microcopy [46]. In this assay, urothelial sections were double-stained using a rabbit antibody to SNX31 and a newly generated mouse monoclonal antibody to uroplakin Ib, followed by incubation with nucleotide-conjugated species-specific secondary antibodies. Close physical proximity of the two secondary antibodies (within 15–20 nm) allows the hybridization of two “linking” nucleotides and the ligase-catalyzed amplification of a concatemeric product that can be detected and quantified digitally [46]. Negative controls, including the replacement of the primary antibodies by pre-immune sera or phosphate-buffered saline or the staining of intestinal epithelial sections, yielded no positive signals. As a positive control, we showed that UPIIIa and Ib, known to form a heterodimer [22]–[24], indeed generated strong PLA signal in the umbrella cells (Fig. 6C, G and K). The urothelial umbrella cells of the Buff mouse (Fig. 6E, I and K), which carries a Vps33a mutation that blocks the fusion of MVBs with lysosomes, are known to accumulate MVBs [44]. Consistent with this, the Buff mouse urothelium showed a tremendous increase in SNX31 staining in its umbrella cells (Fig. 6F vs. D) and a corresponding increase in the PLA signals of SNX31:UPIIIa interaction (Fig. 6J vs. 5H; 5K). In addition to confirming that SNX31 interacted with uroplakins, these PLA results indicated that such SNX31:uroplakin interactions occurred mainly at the MVBs.

**Figure 7:**
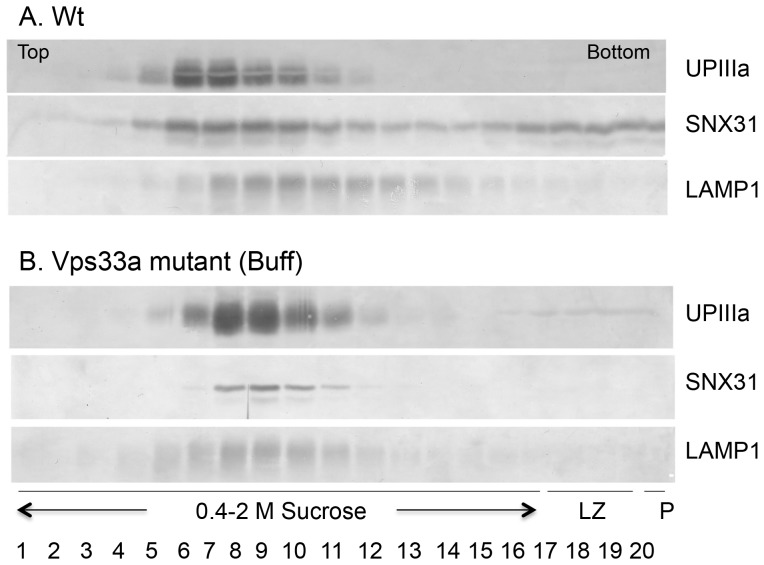
Association of SNX31 with uroplakin IIIa during sedimentation equilibrium centrifugation. Total bladder urothelial proteins from wild type and buff mice were subjected to a flotation analysis on a sucrose density gradient. The total homogenate was loaded at the bottom of a continuous sucrose gradient (0.4 to 2.0 M sucrose), and membranes were separated (fraction 1 represents the lightest and 20 the heaviest). LZ (loading zone) and P (pellet). Following centrifugation, proteins from various fractions were immunoblotted for SNX31, UPIIIa and Lamp1. Note that in normal mouse urothelium (**A**), SNX31 largely co-distributed with the uroplakin peak with the remaining forming a smear, and that, in Buff mouse (**B**), almost all the SNX31 co-floated with UPIIIa.

As another independent way to assess the interactions between SNX31 and uroplakins, we fractionated normal mouse urothelial membranes by flotation equilibrium on a 0.4–2 M sucrose density gradient (Fig. 7). Under this condition, uroplakin IIIa floated as a major peak (1.0–1.1 M sucrose). Some of the SNX31 (30–40%) was associated with this uroplakin peak, with the rest forming a smear in the rest of the gradient presumably reflecting a soluble state. Uroplakins of the Vps33a mutant (Buff) mouse urothelium similarly formed a major peak (1.1–1.2 M sucrose), but, in this case, almost all of the SNX31 co-floated with the uroplakin peak consistent with an enhanced SNX31:uroplakin association as suggested by the pull-down (Fig. 6A and B) and PLA data (Fig. 6H, J and K).

**Figure 8:**
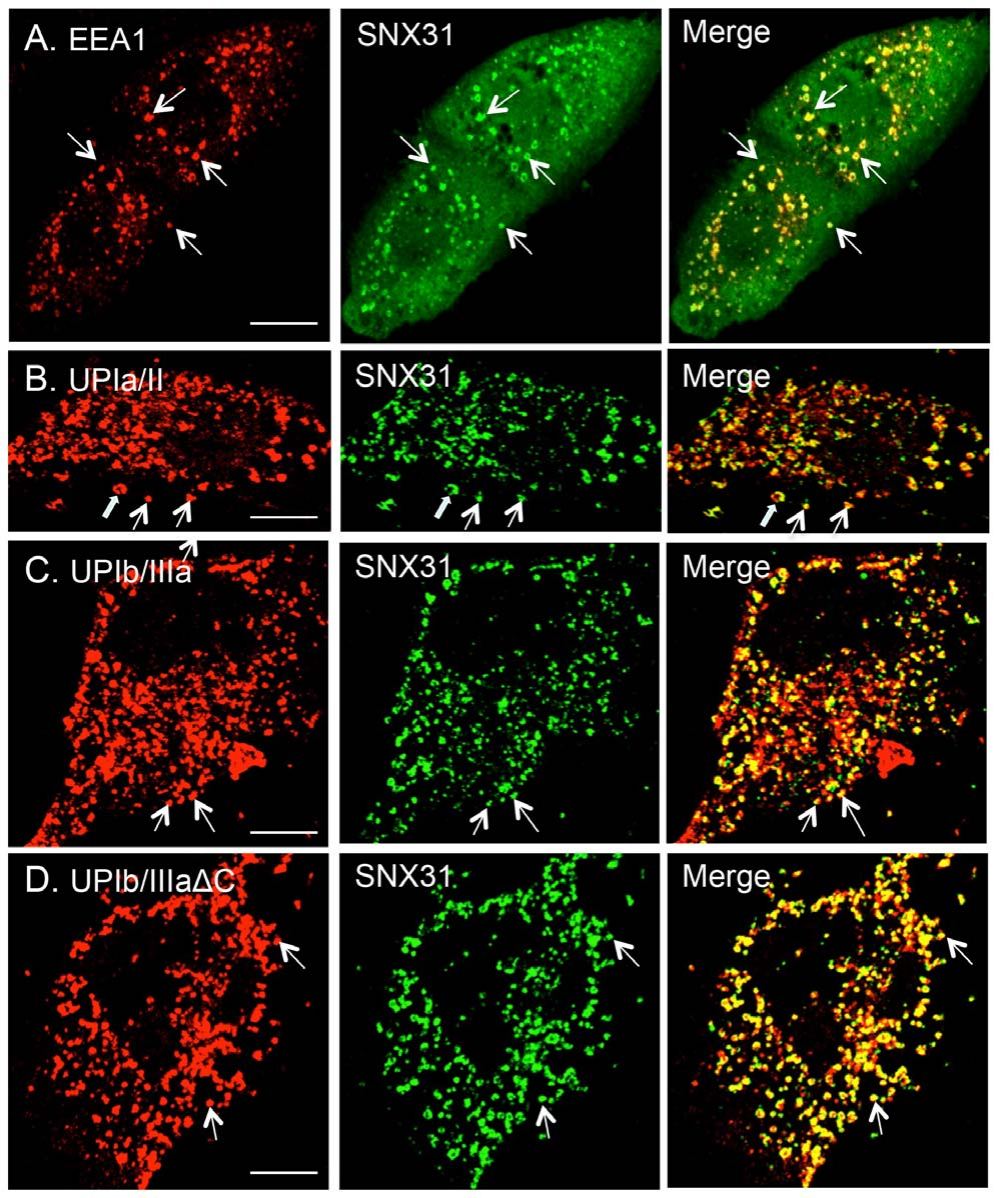
Association of SNX31 with the early endosomes in MDCK cells. MDCK cells were transfected with (**A**) SNX31 alone, or co-transfected SNX31 with (**B**) uroplakins UPIa/II, (**C**) UPIb/IIIa, or (**D**) UPIb/IIIaΔC, fixed 48 hrs post-transfection, and double-stained using antibodies to SNX31 (green) and (**A**) EEA1, (**B**) UPIa of the UPIa/II pair, or (**C** and **D**) UPIb of the UPIb/IIIa pair (red). Merged images are shown to the right. Note in (A) the co-localization of SNX31 with the EEA1-positive early endosomes, in (B and C) the co-localization of SNX31 with the endocytosed uroplakins, and in (D) that the deletion of the cytoplasmic tail of UPIIIa had no effects on the distribution of SNX31. Scale bar  = 10 µm (all panels).

To visualize the subcellular distribution of SNX31 in cultured cells, we studied its expression in transfected MDCK cells (Fig. 8). We found that the ectopically expressed SNX31 bound to cytoplasmic vesicles that were positive for EEA1, an early endosome marker (Fig. 8A), without or with co-transfected uroplakins (Fig 8B–D). This result indicates that the targeting of SNX31 to early endosomes *in MDCK cells* is uroplakin-independent, and that it is probably mediated through the interaction with phospholipids.

### Membrane association of SNX31 decreases in the uroplakin II-knockout urothelium

**Figure 9:**
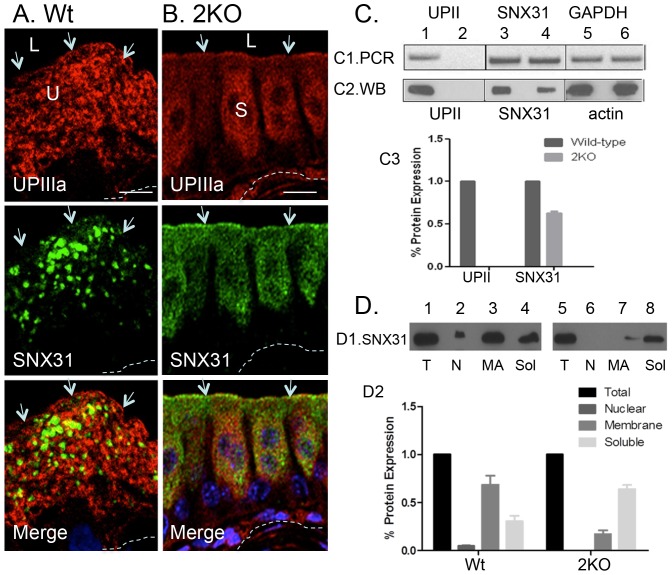
Uroplakin knockout diminishes the membrane association of SNX31. (**A and B**) Immunofluorescent double-staining of uroplakin IIIa (top) and SNX31 (middle) in wild type (**A**; Wt) and UPII knockout (**B**; 2KO) mouse bladder urothelium. Note in (B) the diminished UPIIIa staining and the loss of the characteristic MVB-associated SNX31 staining pattern. (**C**) Effects of UPII-knockout on the mRNA (C1, RT-PCR) and protein levels of SNX31 (C2, Western blot or WB) in wild-type (odd-numbered lanes) and UPII-deficient urothelia (even-numbered). (C1) RT-PCR analyses of (lanes 1–2) UPII, (3–4) SNX31, and (5–6) GAPDH (loading control). (C2) Western blot (WB) analyses of (lanes 1–2) UPII, (3–4) SNX31, and (5–6) actin (loading control). (C3) Quantification of UPII (left) and SNX31 (right) protein amounts normalized against actin, in total mouse protein extracts of the wild-type (dark bars) and UPII KO (grey) mouse bladder urothelia. Values are means±S.D. Note the reduced SNX31 protein levels (∼35% decrease) in the UPII KO mouse (t-test; p<0.01). (**D**) Effects of UPII-ablation on the membrane association of SNX31. (D1) Equivalent amounts of proteins from various fractions of normal (lanes 1–4) and UPII-KO mouse bladder epithelial cells (lanes 5–8) were immunoblotted for SNX31. Lanes (1 and 5; T) total, (2 and 6; N) nuclear, (3 and 7; MA) membrane-associated, and (4 and 8; Sol) soluble proteins. (D2) Quantification of SNX31 protein, as a fraction of the total protein, in the wild-type (left) and UPII KO (right) mouse urothelial extracts. U (umbrella cell); S (superficial cell of the uroplakin-deficient urothelium); other symbols are the same as in Fig. 1. Note the 3–4-fold decrease in the amount of membrane-associated SNX31 in the UPII KO comparing with the wild-type (t-test; p<0.02). Bars  = 10 µm.

We next compared membrane-association status of SNX31 in normal vs. UPII-deficient urothelium (Fig. 9). We described earlier that UPII-knockout leads to (i) a decrease in the protein levels of its partner uroplakin UPIa, as well as the other uroplakin pair UPIb/IIIa (Fig. 9A and B), (ii) the loss of fusiform vesicles, apical urothelial plaques, and the plaque-lined MVBs (Fig. S2); and (iii) a change in the shape of umbrella cells from squamous to cuboidal or even columnar (Figs. 9B and S2; [10], [12]). Immunofluorescent staining showed that the UPII-deficient urothelium lacked the large SNX31-positive MVBs; instead, SNX31 was associated with fine cytoplasmic vesicles (Fig. 9B, middle panel) presumably representing small early endosomes. RT-PCR and immunoblot analyses confirmed that the UPII-knockout urothelium had no detectable UPII mRNA (Fig. 9C1, lanes 1 and 2) or protein (Fig. 9C2, lanes 1 and 2), but a normal level of SNX31 mRNA (Fig. 9C1, lanes 3 and 4; and Fig. 9C3), indicating that SNX31 transcription was not affected by UP knockout. However, the SNX31 protein level was reduced by about 35% (Fig. 9C2, lanes 3 and 4; and Fig. 9C3) suggesting that UP knockout led to a destabilization of SNX31. Finally, although in normal urothelium 60–70% of SNX31 was membrane-associated (Fig. 9D1, lanes 1–4; and Fig. 9D2), less than 15–20% of SNX31 was membrane-bound in UPII-deficient mouse urothelium (Fig. 9D1, lanes 5–8; and Fig. 9D2), suggesting that, in the *in vivo* urothelial umbrella cells, uroplakin deficiency led to the destablilization of the membrane-association of SNX31 (see Discussion).

### SNX31 binds to PtdIns3P in a PtdIns3P kinase-dependent manner

**Figure 10:**
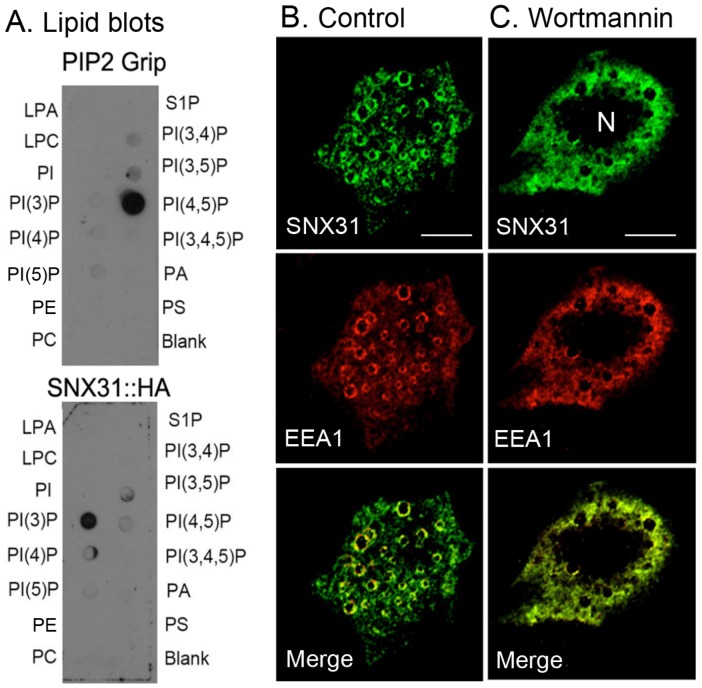
SNX31 preferentially binds to PtdINs(3)P. (**A**) Immunoaffinity-purified PIP2:GST and SNX31:HA fusion proteins were used to overlay PIP strips (Echelon) spotted with 100-pmol of various phosphoinositides. The bound proteins were detected with antibodies to GST and HA epitopes. (**B**) MDCK cells were transfected with SNX31 and stained using affinity-purified anti-SNX31 antibodies (top panel) and EEA1 antibody (middle), with merged images at the bottom. (**C**) Cells were treated with wortmannin (100-nM, 30 min), a PtdIns(3)P-kinase inhibitor. Abbreviations are: N (nucleus), LPA (lysophosphatidic acid), LPC (lysophosphacholine), PA (phosphatic acid), PC (phosphatidylcholine), PE (phosphatidyl-ethanolamine), PS (phosphatidylserine) and S1P (sphingosine-1-phosphate). Note that PtdIns(3)P-K inhibition abolished the association of both SNX31 and EEA1 with the early endosomal membranes, leading to a diffuse, cytoplasmic staining pattern. Bars  = 10 µm.

The ability to bind distinct phosphoinositides through their PX domain is a hallmark of the SNX proteins and allows SNXs to target their cargoes to specific cellular membranes [39], [47]. To determine the lipid-binding specificity of the SNX31, we transfected 293T cells with a cDNA encoding SNX31 that was C-terminally tagged with HA. We then affinity-purified this protein and used it to overlay a nitrocellulose membrane with spots containing 100 pmol of various lipids (Fig. 10A). The lipid binding-specificity of SNX31:HA and the positive control, ‘PtdIns(4,5)P_2_ grip’, was then detected using antibodies against the HA antigen or GST, respectively. We found that SNX31 preferentially bound PtdIns(3)P (Fig. 10A). In another experiment, we found that SNX31 co-localized precisely with EEA1 in the vesicular endosomes of transfected MDCK cells (Fig. 10B; also see Fig. 8A). However, this association was abolished by wortmannin (Fig. 10C; [47], [48]). Hence, the association of SNX31 with early endosomes is largely dependent on PtdIns(3)P.

## Discussion

### SNX31 is a highly urothelium-enriched and differentiation-dependent sorting nexin

Sorting nexins are a family of PX (phox-homology) domain-containing proteins that link various protein cargoes to endosomes. These proteins usually have a broad tissue-distribution (see Fig. 1A, e.g., for the wide tissue-distribution of the closely related SNX17) performing general functions in membrane trafficking, cell signaling and organelle motility [39], [40], [49], [50]. The urothelium-specificity (Figs. 1A and S1) and differentiation-dependent expression of SNX31 (Fig. 1B, D and E) is quite exceptional, suggesting that it must perform an important function in terminally differentiated urothelial cells.

### Possible roles of *Get1* gene in the co-regulation of uroplakin and SNX31 genes

Andersen and co-workers showed that the ablation of the *Get1/Grhl3* gene that encodes grainyhead, a transcription factor that binds to the promoter of UPII gene, led to the down regulation of not only genes encoding uroplakin II (7.56 fold reduction) and other uroplakins, but also the SNX31 gene (30.17 fold; [51]), suggesting that Get1 may contribute to the co-expression of the SNX31 and uroplakin genes in the urothelial umbrella cells.

### SNX31 binds to uroplakin proteins in urothelial umbrella cells but not in transfected MDCK cells

Our data indicate that SNX31 interacts with uroplakins, based on immunoprecipitation (Fig. 6A and B), proximity ligation assay (Fig. 6H, J and K), EM co-localization on the limiting membranes of MVBs and ILVs (Fig. 3), and co-floating during sucrose gradient centrifugation (Fig. 7). It is interesting that, in extracts of the *in vivo* urothelium, SNX31 co-immunoprecipitated with uroplakins Ib and IIIa that form the UPIb/IIIa pair (Fig. 6). Since UPIb has very little cytoplasmic domain [5], [18], [20]), we hypothesize that SNX31 pulled down UPIIIa of the (UPIb/IIIa) heterodimer via UPIIIa's cytoplasmic tail.

Although urothelial cells cultured in the presence of 3T3 feeder cells can stratify and differentiate [41], their differentiation deviates significantly from that of *in vivo* urothelium [52]. Unlike *in vivo* urothelium whose uroplakins are fully assembled to form 2D crystals of 16-nm particles covering the apical urothelial surface, the uroplakins in cultured urothelial cells do not form 16-nm particles let alone 2D crystals ([9], [24], [41], [52], [53]; cf. [54], [55]). It is therefore perhaps not surprising that even though uroplakins of the *in vivo* urothelium can co-immunoprecipitate with SNX31 (Fig. 6), those of cultured urothelial cells (or uroplakin dimer-cotransfected MDCK cells) fail to do so (data not shown) – possibly due to a lack of certain conformation or secondary modifications that are unique to uroplakins of *in vivo* urothelium.

### Functional diversities of the FERM-containing sorting nexins

SNX31, SNX17 and SNX27 form a small subgroup of sorting nexins sharing a 4.1/ezrin/radixin/moesin (FERM)-like domain [56]. Collins and co-workers showed recently that the FERM-like domain of SNX17 and SNX27 can recognize the NPxY motif *in vitro*, and that all three sorting nexins bind Ras GTPase [56]. While these three sorting nexins are structurally related, they seem to target different classes of protein cargos. For example, the FERM- and/or C-terminal domain of SNX17 can bind the NPx(F/Y) motif in the cytoplasmic tails of several membrane cargo proteins including P-selectins [57], LDL receptors [58], and the amyloid precursor protein [59]. Functionally, SNX17 promotes recycling and suppresses the degradation of its membrane cargo proteins [60], [61]. In the case of SNX27, it contains an additional PDZ domain that targets proteins containing a PDZ-binding motif, including beta-Pix (p21-activated kinase-interactive exchange factor) [62] and multidrug resistance-associated protein 4 (MRP4/ABCC4) [63]. Functionally, SNX27 in general enhances the recycling of plasma membrane (cf. [63]). Finally, we show here that SNX31 may facilitate multivesicular body-mediated uroplakin degradation in urothelial umbrella cells (see below). Together, these data indicate that, despite their structural relatedness, these three sorting nexins target different classes of cargo proteins and perform diverse tissue-specific and cellular context-dependent functions.

### Possible roles of SNX31 in the uroplakin-degrading multivesicular vesicles: a model

**Figure 11:**
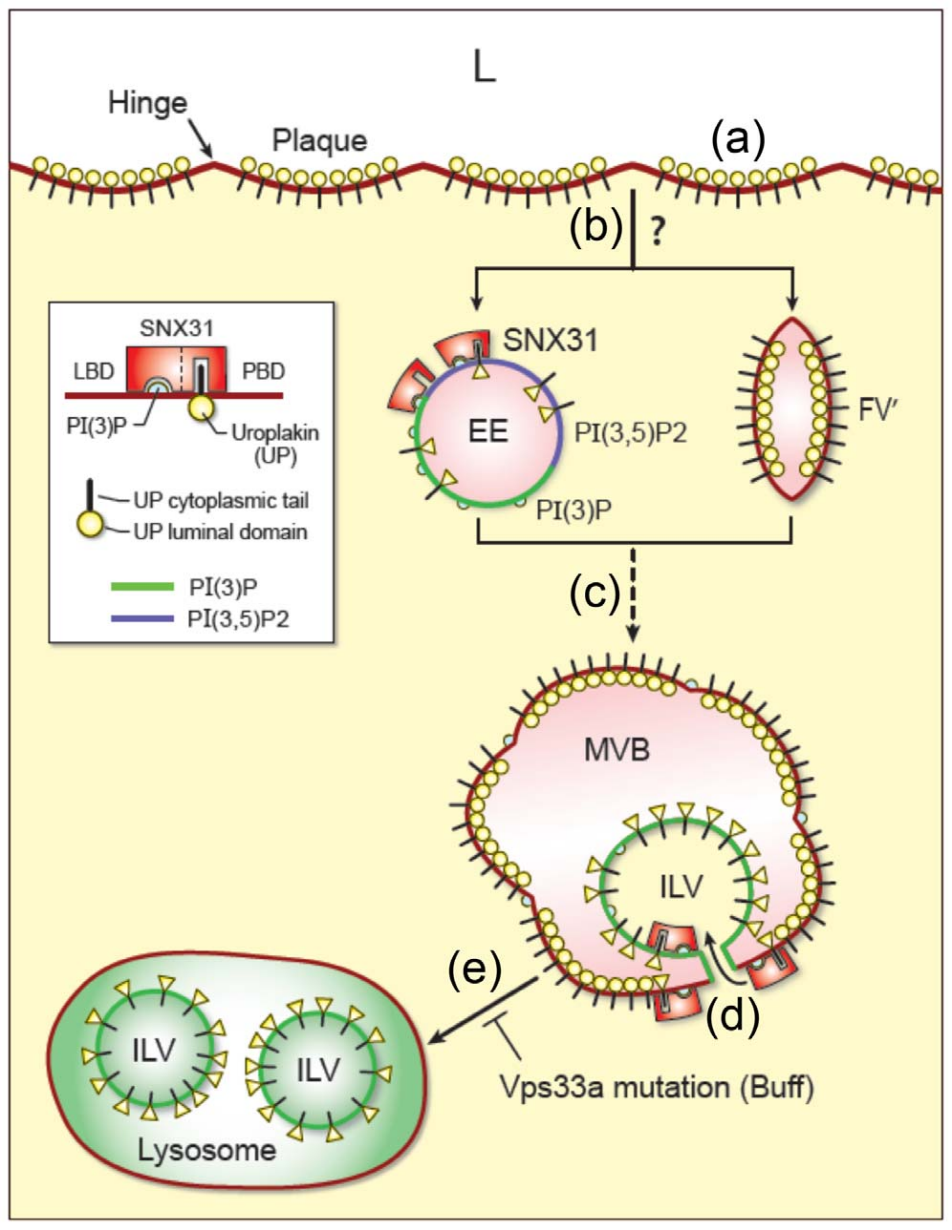
A model for the possible roles of SNX31 in the endocytic degradation of uroplakins. The model illustrates that: (**a**) The apical surface of mouse bladder urothelium is lined by urothelial plaques consisting of hexagonally packed, 2D crystals of 16–nm uroplakin particles (lollipods). (**b**) Apical uroplakins are endocytosed by a poorly understood (hence question-marked), clathrin- and caveolin-independent process leading to the possible formation of small, early endosomes (EE) or endocytic ‘fusiform-like vesicles’ (‘FV’ to distinguish them from the regular, exocytic fusiform vesicles, FV; see Figure 8 in [45]). Although the binding of SNX31 to some EE cannot be excluded, such structures have not yet been detected. (**c**) The multivesicular bodies (MVBs) of bladder urothelial umbrella cells are highly specialized as they are lined by SNX31-associated uroplakin plaques. (**d**) SNX31 and uroplakins co-enter into the intraluminal vesicles (ILVs). It is hypothesized that the binding of SNX31 to the cytoplasmic tail of UPIIIa can cause the uroplakin particles to dissociate from the plaque and to collapse (changing the shape of the luminal 16-nm particles from circle to triangle in the diagram) thus facilitating the invagination of the uroplakin-containing membrane to form ILVs. (**e**) MVBs fuse with lysosomes for uroplakin degradation [35], [36]; this process is blocked in Buff mouse carrying a Vps33a mutation. This model depicts the possible roles of SNX31 in facilitating the invagination of the MVB-associated uroplakins enabling them to enter into the intraluminal vesicle compartment. See Discussion for details. EE (early endosome), FV' (endocytic ‘fusiform vesicles-like vesicles’), ILV (intraluminal vesicle), L (lumen), MVB (multivesicular vesicle), SNX31-LBD (lipid binding PX domain), SNX31-PBD (protein-binding domain) and UP (uroplakin).

Figure 11 is a schematic diagram that provides a unifying model tying together all our data, and proposes a possible SNX31 function. As indicated in the diagram, the apical surface of umbrella cell is covered by uroplakin plaques (*step a*), which are endocytosed by a clathrin- and caveolin-independent mechanism (*step b*; [31]). It is unclear, however, whether this proceeds via the formation of small early endosomes (EE) or large fusiform-like vesicles (FV'; hence the question mark at *step b*; unpublished data).

Existing evidence suggests that MVBs are involved in uroplakin degradation (*step c*). (i) MVBs in umbrella cells are lined with urothelial plaques morphologically indistinguishable from the apical plaques (Figs. 3 and 4; [35], [64], [65]). (ii) Lectin-, peroxidase- as well as biotin-tagged apical uroplakins are delivered to MVBs (Fig. 5; also see [28], [35], [36]). (iii) Vsp33a mutation, which blocks the fusion of MVBs to lysosomes, leads to the accumulation of MVBs and hindered uroplakin degradation [44]. Finally, (iv) overexpression of MAL, which facilitates the fusion of the exocytic uroplakin vesicles with the apical surface, stimulates uroplakin exocytosis and subsequent compensatory endocytosis, which in turn leads to a massive accumulation of MVBs and a decrease in uroplakin content [45]. Since proteins remaining on the limiting membranes of MVBs are known to be primarily recycled while those incorporated into the ILVs are largely destined for lysosomal destruction [66], [67], most of the uroplakins sorted into the SNX31-positive ILVs are probably destined for lysosomal degradation. However, one cannot rule out the possibility that some of the ILV-associated uroplakins are released to the extracellular space via the exosome pathway because small amounts of uroplakins have been detected in the urine-associated exosomes [68].

Our data indicate that SNX31 can bind to both uroplakin proteins (Fig. 6) which are mainly associated with apical surface and MVB, as well as the PtdIns(3)P-lipids (Fig. 10), which are associated with early endosomes, MVBs and their ILVs [66], [69]. The fact that MVBs (and ILVs) are the only organelles that contain both SNX31 ligands (uroplakins and PtdIns(3)P) may account for SNX31's preferential binding to MVBs (Figs. 1 and 5; *step c* in Fig. 11).

An interesting feature of SNX31 is that it has an ESCRT-like domain. Protein sorting to MVBs and their entry into ILVs requires ubiquitination and ESCRT machinery [66], [70]. The ESCRT complexes have ubiquitin-binding domains (UBDs) that can recognize the ubiquitinated cargo for it's sorting into the ILVs of the MVBs, followed by lysosomal degradation [71]. Although the C-terminus of UPIIIa is the major cytoplasmic domain of the uroplakin complex, we could not detect UPIIIa ubiquitination (data not shown). It will be interesting to determine in future studies whether uroplakin sorting to the MVBs occurs via a ubiquitin- and ESCRT-independent mechanism [70], [72], [73]. Alternatively, it is possible that the ESCRT machinery can recognize the ubiquitin-like domain of the SNX31, thus allowing the endocytosed uroplakin vesicles to target to MVBs and enter into ILVs (*step d*).

The invagination of the MVB-associated uroplakins to form small ILVs (step d) requires a significant bending of the rigid-looking urothelial plaques, which presents a major energetic barrier [37]. This invagination process can be greatly facilitated, however, if the uroplakin particles can be disassembled. Existing evidence indicates that uroplakin particles can indeed dissociate and undergo major conformational changes in response to mechanical stress or protein-protein interactions. First, we showed earlier by quick-freeze deep-etch electron microscopy that the hinge areas interconnecting the neighboring uroplakin plaques are not just particle-free ‘regular-looking membranes’, as was thought earlier. Rather, these areas contain numerous dissociated and partially collapsed uroplakin particles [7] and are, like the plaques *per se*, detergent-insoluble suggesting that these are also uroplakin-containing membranes [74]. Moreover, when isolated apical urothelial membranes reseal *in vitro* to form an outside-out vesicle, the plaques (normally 600–900 nm in diameter containing 1,400–3,000 particles) can break up into smaller ones (100–400 particles) in response to an increased membrane curvature. The newly formed hinge areas that separate the smaller plaques again contain dissociated and collapsed uroplakin particles morphologically similar to those found in the hinge areas of normal bladder urothelium [28]. These data suggest that the dissociation and collapse of uroplakin particles may be the normal mechanism underlying hinge formation [7]. Second, we showed by cryo-electron microscopy that binding to the extracellular domain of uroplakins by a single protein, i.e., the bacterial lectin FimH, can induce uroplakin particle to undergo global conformational changes [75]. Third, as mentioned earlier, uroplakins synthesized by cultured urothelial cells do not form 16-nm particles [9], [24], [52]. Taken together, these data suggest that the structure of the 16-nm uroplakin particles in 2D plaques is quite flexible as they can readily dissociate and undergo major conformational changes or even collapse in response to physical stress, ligand binding and cell culture conditions. Based on these data, we hypothesize that SNX31, once bound to the cytoplasmic tail of the MVB-associated UPIIIa, may cause the uroplakin particle located at the edge of a plaque to dissociate from the plaque and to collapse, thus facilitating the invagination of the uroplakin-containing membranes to form ILVs that are destined for lysosomal degradation (*step e*). We further hypothesize that continued binding of SNX31 to the cytoplasmic tail of uroplakins is required in order to maintain a collapsed uroplakin configuration in early ILVs (Fig. 11, *step d*). Additional work is needed to test the validity of this model.

## Materials and Methods

### Mice

This study was carried out in strict accordance with the recommendations in the Guide for the Care and Use of Laboratory Animals of the National Institutes of Health. The protocol was approved by the Committee on the Ethics of Animal Experiments of NYU Langone Medical Center (protocol #'s 090905-01 and -02). All surgery was performed under sodium pentobarbital anesthesia, and all efforts were made to minimize suffering. All studies were performed with the approval of the NYU Medical Center Institutional Animal Care and Use Committee (IACUC).

### Tissue distribution of SNX31

Total RNAs were isolated using the RNeasy midi kit (Qiagen cat 75144), following the manufacture's instruction, from 1–3 g of mouse and bovine tissues. Mouse cDNA was synthesized by RT-PCR using a Superscript first-strand system (Invitrogen) with the primers: RTSNX31 Fwd and RTSNX31 Rev; length: 226 bp; cycle protocol: 95C 10 min; 95C 30 s, 58C 30 s, 72C 30 s 25 cycles; 72C 10 min. Bovine RNA was separated on a 1% agarose gel, transferred to a Hybond nitrocellulose membrane (Amersham Biosciences) and probed with a *bSNX31* specific probe labeled with ^32^P, using the Rediprime II DNA-labeling kit (Amersham Biosciences).

### Generation and characterization of rabbit antibodies to SNX31

Rabbit antisera were raised against synthetic peptides corresponding to (i) amino acid residues 423–439 (CKRSEGDYVWDTLMEEGL), the C-terminus of the mouse SNX31; (ii) amino acid residues 387–402 (C-SPEMQIEVPEQGRSKK) located near the C-terminus of the bovine SNX31. The antibodies were affinity purified using the original peptide antigen and shown to be mono-specific when used to immunoblot the total proteins of mouse and bovine urothelia, or the total proteins of 293t cells that had been transfected using cDNA's encoding mouse or bovine SNX31.

### Fractionation of urothelium or transfected 293T cells

Total cell extracts of bovine bladder epithelial cells were fractionated by centrifugation (60 min at 10000×g) to produce a pellet containing nuclei and all cellular membranes and a soluble fraction [21], [43]. Equivalent amounts of samples were loaded on SDS polyacrylamide gels (17%) followed by immunoblotting using an anti-SNX31 antibody. Transfected 293T cells or mouse and bovine bladder epithelial cells were washed in PBS pH 7.4 and lysed for 10 min at room temperature with RIPA buffer (1% NP-40, 0.5% DOC, 0.1% SDS, in 150 mM NaCl, 50 mM Tris (pH 7.5)), supplemented with protease inhibitor cocktail (Roche, Germany) [45]. Protein quantification was accomplished by using the BCA protein assay (Thermo Scientific, USA) and BSA used as a protein standard. Equal amounts of protein were separated by SDS polyacrylamide gel electrophoresis in 17% acrylamide gels and electroblotted onto a Transblot nitrocellulose membrane (BioRad, USA). Membranes were blocked in PBS pH 7.4 with 5% non-fat dry milk and probed with monoclonal anti-HA antibody (Sigma). Horseradish peroxidase-conjugated anti-mouse or anti-rabbit immunoglobulin G was used as secondary antibodies (Sigma). Immunoreactive bands were detected by chemiluminescence (ECL).

### Construction of pcDNA3:HA vector

In order to construct a C-terminal HA-tagged pcDNA3 vector, primers CHAF/CHAR were boiled for 5 min in annealing buffer (10 mM Tris HCl, pH 7.5–8.0, 50 mM NaCl) and cooled to room temperature to get annealed through their complementary sequences. The vector pcDNA3 (Invitrogen) was digested by ApaI (NEB). The annealed double-stranded oligonucleotide was ligated into the digested vector in the molar ratio of 3:1 by T4 ligase (NEB) at 16°C overnight. The recombinant DNA was transformed into competent bacteria host cell, Top10. Confirmation polymerase-chain reaction was performed with the primers: 5′TACGATGTTCCAGATTAC3′ and 5′ GTAAAGCACTAAATCGGAA3′ and sequenced. Mouse SNX31 cDNA was then used as a template to amplify the mSNX31 ORF sequence with the primers: CFLF/CFLR. The C-terminal HA tagged pcDNA3 vectors were digested with HindIII and XbaI (NEB) and ligated to the amplified mSNX31 PCR products. Top10 competent *E. coli* was transformed with recombinant plasmids. Positive clones were subsequently sequenced.

### Transfection

293T and MDCK cells were cultured in MEM medium (Cellgro, Mediatech Inc.) supplemented with 1% PS (Gibco) and 10% FBS (Fisher/Hyclone), at 37°C in a humidified 5% CO_2_ chamber. 80% confluent growing suspensions seeded 18 h before were transfected with the PolyJet reagent (SignaGen Laboratories). A mixture of DNA, PolyJet 1:3 in serum free DMEM was added to cells after 10 min of incubation at room temperature. Cells were analyzed 42 h post transfection [45].

### Immunofluorescence microscopy

Paraffin sections of urinary bladders from 8 to 12 week-old mice were de-paraffinized, rehydrated, and microwaved to expose the antigenic sites. Transfected MDCK cells were cultured on cover slips, fixed in 4% paraformaldehyde solution in PBS and permeabilized in 1% Triton-X 100. All samples were then incubated with primary antibodies: monoclonal anti-UPIII (1:100) (AU1); monoclonal anti-UPII (1∶100) (S3045); monoclonal anti-Ib; monoclonal anti-HA (1∶500) (SIGMA); monoclonal anti-EEA1 (1∶500) (BD Biosciences; San Jose, CA): polyclonal-EEA1 (1∶50) (Santa Cruz Biotechnology, Inc., Santa Cruz, CA); polyclonal anti-SNX31 (1∶20); polyclonal anti-biotin (1∶100 polyclonal anti-LAMP1 (1∶100) (BD Biosciences; San Jose, CA); polyclonal anti-clathrin (1∶100) (BD Biosciences; San Jose, CA); polyclonal anti-caveolin-2 (1∶100) (BD Biosciences) for 1 h at 37°C (tissue preparation) or RT (transfected cells), followed by washing with PBS and incubation with either FITC or Texas red-labeled donkey: anti-rabbit IgG (1∶200); anti-mouse IgG (1∶200) or anti-goat IgG (1∶200) for 45 min at room temperature. The sections and cover slips were examined with a Zeiss LSM 510 confocal microscope using the 488 nm and 561 nm lasers and a 63× objective lens. Filters used were 500–530 nm (GFP, FITC and Alexa488) and 550–650 nm (Rape, RFP and Alexa568).

### Electron microscopy

For ultrastructural study, mouse bladder urothelial samples were high pressure frozen in a Balzers HPM 010 apparatus, and freeze-substituted first with acetone containing 2% OsO_4_ and then with 100% acetone in a Leica AFS apparatus. The samples were then embedded in Epon, and ultrathin (60 nm) sections were cut and counterstained with lead citrate and uranyl acetate [28].

For EM localization using the Lowicryl procedure, mouse bladders were excised and fixed for 4 h in 0.1 M sodium cacodylate buffer (pH 7.4) containing 3% (w/v) paraformaldehyde, 0.1% glutaraldehyde (v/v) and 4% sucrose. The samples were embedded in Lowicryl K4M (Polysciences, Inc., Warrington, PA) and polymerized under UV light (360 nm) at −35°C. For single labeling, ultra-thin sections were incubated with primary antibodies (AU1 for UPIII and 1∶10 for anti-SNX31) at room temperature for 2 h and then 4°C overnight. Protein A-conjugated 15-nm gold particles (Cell Microscopy Center, University Medical Center Utrecht, 35584 CX Utrecht, The Netherlands) were used as secondary antibody. Double labeling was performed as previously described, with minor modifications by using 15-nm protein A gold particles for SNX31 and 5-nm protein gold for uroplakin III [45].

Ultrathin sections of Lowicryl K4M embedded biotinylated mouse bladder were cut, mounted on formvar-carbon coated nickel grids. After incubation with Nanogold conjugated streptavidin (1∶50, Nanoprobes, Yanphank, NY), silver enhancement was performed in the dark for 8 minutes using HQ Silver Enhancement kit (Nanoprobes). Grids were stained with 3% uranyl acetate and examined under Philips CM-12 electron microscope and photographed with a Gatan (4kx2.7k) digital camera.

For cryoEM localization, mouse bladders were fixed with 4% paraformaldehyde in PBS for 1 h, rinsed in PBS, embedded into 10% gelatine blocks, cryoprotected by incubation in cold 2.3 M saharose and frozen in liquid nitrogen. Cryosections (55 nm thick) were cut with Leica FCS cryo-ultramicrotom at -120°C and collected on gold EM grids. Primary rabbit polyclonal antibodies against AUM (diluted 1∶10,000) were incubated overnight at 4°C, washed in PBS, and incubated with goat anti-rabbit secondary antibodies, conjugated with 10 nm colloidal gold (Sigma, diluted 1∶40) at room temperature for 1.5 h. Cryosections were counterstained with methyl cellulose/uranyl acetate solution and examined with Philips CM100 transmission electron microscope, running at 80 kV.

### Floatation analysis of the mouse urothelium

Four wild type (C57/B6) or VPS33a mutant (Buff) mouse urothelia were homogenized in 200 µl homogenization buffer (HB: 25 mM Hepes-KOH pH 7.3, 1 mM DTT, 0.2% M sucrose, 1 X Roche Complete Mini protease inhibitor cocktail tablets) with a 1 ml Wheaton Dounce homogenizer. A 400 µl aliquot of 3.0 M sucrose in HB lacking the 0.25 M sucrose were added to the 2 homogenates and mixed thoroughly resulting in a sucrose concentration of about 2.08 M. Each 0.6 ml sample was overlaid by a 3.4 ml continuous 0.4 M to 2.0 M sucrose gradient (total 4 ml) in a SW60 tube. The samples were spun for 20 hrs at 200,000×g (45,000 rpm) at 4°C. Following centrifugation, a series of 200 µl fractions were collected from the top to the bottom of the tube (20 fractions: 1 to 17 =  sucrose gradient, 18–20 =  loading zone). The pellet was re-suspended in 200 µl of HB buffer (fraction 21). A series of 50 µl aliquots (which contains the material corresponding to 1 urothelium) from the 21 fractions were analyzed by PAGE (10/15% gel) and WB for the distribution of the proteins indicated in the Sures. The protein and sucrose density distributions throughout the gradients were determined with the Bio-Rad protein assay and with a Bausch and Lomb refractometer, respectively.

### Immunoaffinity purification

Monoclonal anti-HA agarose conjugate clone HA-7 beads (Sigma) were used to immunoaffinity-purify mSNX31:HA. Beads were packed in empty chromatography column with PBS pH 7.4. Column was then washed 3x with Glycine-HCL 0.1 M pH 2.5 (5x the column volume), followed by three sequential aliquots of PBS pH 7.4 (5x the column volume). Total protein lysate was loaded into the column and incubated overnight at 4°C. The flow through was collected and the column washed with PBS pH 7.4 till OD_280nm_ reached 0.01. mSNX31:HA was subsequently eluted with 8 aliquots (column volume) of Glycine-HCL 0.1 M pH 2.5 into vials containing 1 M of Tris HCl pH 8.0, in order to immediately neutralize the pH. The column was promptly regenerated by washing with Glycine-HCL 0.1 M pH 2.5 and PBS pH 7.4.

### Lipid binding specificity assay

PIP strips spotted with 100 pmol of different phospholipids were purchased from Echelon Research Laboratories. Membranes were blocked with PBST with 3% (w/v) BSA fatty acid free (Sigma, A7030) for 1 h, at room temperature, with gentle agitation. Blocked membranes were incubated with 0.5 ug/ml of mSNX31:HA or PIP2Grip (positive control from Echelon Research Laboratories) in PBST 3% (w/v) BSA fatty acid free, for 2 h at RT and then washed in PBST 6x over 30 min. Membranes were sequentially incubated with anti-HA (Sigma) or anti-GST (Sigma), respectively, at 1∶5000 dilution in PBST 3% (w/v) BSA fatty acid free, for 50 min. Washes were performed as previously described. Membranes were incubated with secondary mouse HRP antibody (Sigma), 1∶500 for 45 min also in PBST 3% (w/v) BSA fatty acid free. Following final washing, enhanced chemiluminescence was used to detect binding of HA or GST tagged proteins to phospholipids.

### Wortmannin treatment

MDCK cells were transfected with mSNX31 and 48 h post-transfection incubated for different periods of time at 37°C in the presence or absence of 100 nM of wortmannin (SIGMA). Cells were then washed in PBS pH 7.4, fixed in 4% paraformaldehyde and permeabilized in 1% Triton X-100 to pursuit with an immunofluorescence assay.

### Biotinylation

In order to biotinylate apical membrane proteins, Swiss Webster female 8–12 weeks old mice were anesthetized with isofluorane, instilled with 75 µl of PBS, and massaged to induce urination. One more wash was performed with 75 µl of biotinylation reagent and the bladder voided again. 75 µl containing Biotinylation sulfo-long chain biotin reagent at 1 mg/ml (2.1 mM) and 75 µl of PBS pH 8 were then added for 15 min. Bladder was voided and sequentially washed with 75 µl of PBS containing 5 mM of lysine. Mice were kept for 0, 30, 90 min and 3 hrs. Bladder was then removed and fixed in 3.7% formaldehyde in PBS pH 7.4 for 2 hr at RT, and processed for paraffin embedding; or fixed with 3% paraformaldehyde, 0.1% glutaraldehyde (v/v) and 4% sucrose in 0.1 M sodium cacodylate buffer, and embedded with Lowicryl K4M for immuno-electron microscopy [45]. Immunofluorescent staining for biotin was then performed with antibody Anti-Biotin (Rockland Immunochemicals, Inc) (TOPO3 for nuclear staining).

### Co-immunoprecipitation assay

For immunoprecipitation of SNX31 and UPIIIa an activated aminolink plus resin (Thermo Scientific, Pierce, 26149) was pre-crosslinked with 60 µg of immunoaffinity-purified SNX31 antibody. Total urothelial cells were lysed in RIPA buffer (50 mM Tris-HCl pH 7.4, 150 mM NaCl, 0.1% SDS, 0.5% DOC, 1% NP40, supplemented with Roche Proteinase inhibitor) and 300 µg of total proteins were incubated overnight with either: control agarose beads (non activated), quenched resin (200 µl of quenching buffer were added to antibody coupling resin instead of SNX31 antibody) or with aminolink activated resin (all 25 µl beads). The beads were sedimented (5000 g, 2 min) and washed for 6x with cold lysis buffer. The antigens were then eluted with 0.2% SDS sample buffer, analyzed in denaturing-reducing SDS-PAGE and detected with respective antibodies.

### *In situ* proximity ligation assay (*in situ* PLA)

Paraffin sections of wild-type mouse bladder (0.5 µm) were blocked in 1% Fish gelatin in PBS, incubated overnight with the same blocking solution supplemented with antibodies for: UPIb (1∶50), UPIIIa (1∶5) and SNX31 (1∶200). Samples were thereafter subjected to *in situ* PLA using Duolink Detection kit (Olink, Bioscience, Uppsala, Sweden). Briefly, samples were incubated with PLA probes (secondary antibodies) and the detection was done according to manufacturer's protocol. Slides were mounted with Duolink Mounting media with DAPI and analyzed using a Zeiss LSM 510 confocal microscope [46]. The cell images were exported using LSM software in TIF format for further analysis and the determination of signals performed with the Duolink Image Tool. Signals were normalized with the control signals. Quantifications are given as a number of signals per umbrella cell.

## Supporting Information

Figure S1 **Tissue distribution of SNX31 in bovine tissues**: 20 µg of total RNAs from bovine urothelium and other tissues were separated by agarose gel electrophoresis and probed for (**A**) SNX31, (**B**) glyceraldehyde phosphate dehydrogenase (G), and (**C**) the 28S and 18S ribosomal RNAs (ethidium bromide (EB) staining; as a loading control). Lanes are: (1) bladder, (2) kidney cortex, (3) kidney medulla, (4) lung, (5) spleen, (6) esophagus, (7) stomach, (8) intestine, (9) brain, (10) liver, and (11) skeletal muscle. Note the bladder urothelium-specific expression of SNX31.
(TIF)

Figure S2 **Uroplakin II-deficient mouse urothelium lacks the uroplakin-plaque lined multivesicular bodies.** Note in (**A to C**) a lack of fusiform vesicles, apical uroplakin plaques, and multivesicular bodies, that are typical of normal urothelial umbrella cells (cf. Figs. 3A and 4A; [11], [12]). Rather, the cytoplasm of UPII-deficient superficial cells is filled with small MAL-positive vesicles (SV) that are involved in delivering the remaining UPIb/IIIa uroplakin pair to the apical surface [45]. Note in (**D and E**) that, despite the lack of MVBs, late endosomes (LE) and lysosomes (Lys) are clearly identifiable. Magnification bars  = 1 µm in A-C, and 0.2 µm in D and E.
(TIF)
