# Supplementary figures and images for: SNX31: A Novel Sorting Nexin Associated with the Uroplakin-Degrading Multivesicular Bodies in Terminally Differentiated Urothelial Cells
Source: PLoS One. 2014 Jun 10;9(6):e99644. doi: 10.1371/journal.pone.0099644 (PMC4051706; doi:10.1371/journal.pone.0099644)

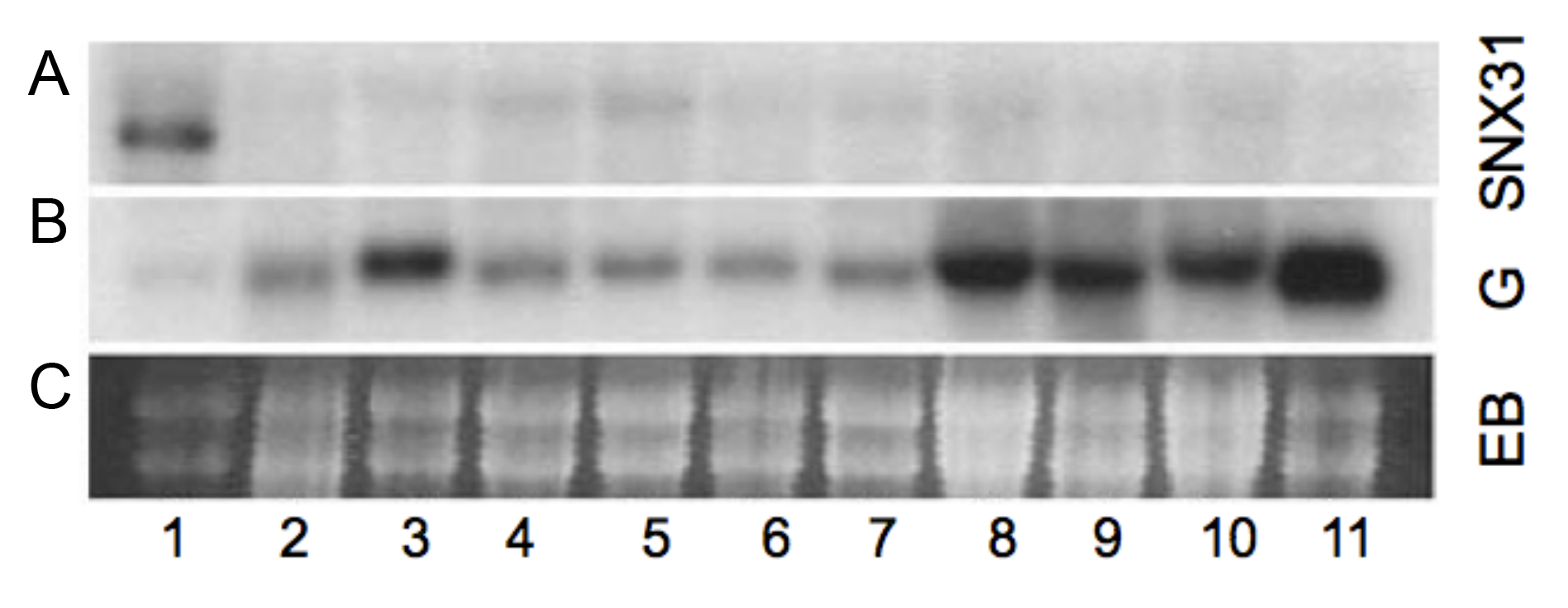

Supplement: Figure S1 — Tissue distribution of SNX31 in bovine tissues: 20 µg of total RNAs from bovine urothelium and other tissues were separated by agarose gel electrophoresis and probed for (A) SNX31, (B) glyceraldehyde phosphate dehydrogenase (G), and (C) the 28S and 18S ribosomal RNAs (ethidium bromide (EB) staining; as a loading control). Lanes are: (1) bladder, (2) kidney cortex, (3) kidney medulla, (4) lung, (5) spleen, (6) esophagus, (7) stomach, (8) intestine, (9) brain, (10) liver, and (11) skeletal muscle. Note the bladder urothelium-specific expression of SNX31. (TIF) [file pone.0099644.s001.tif]

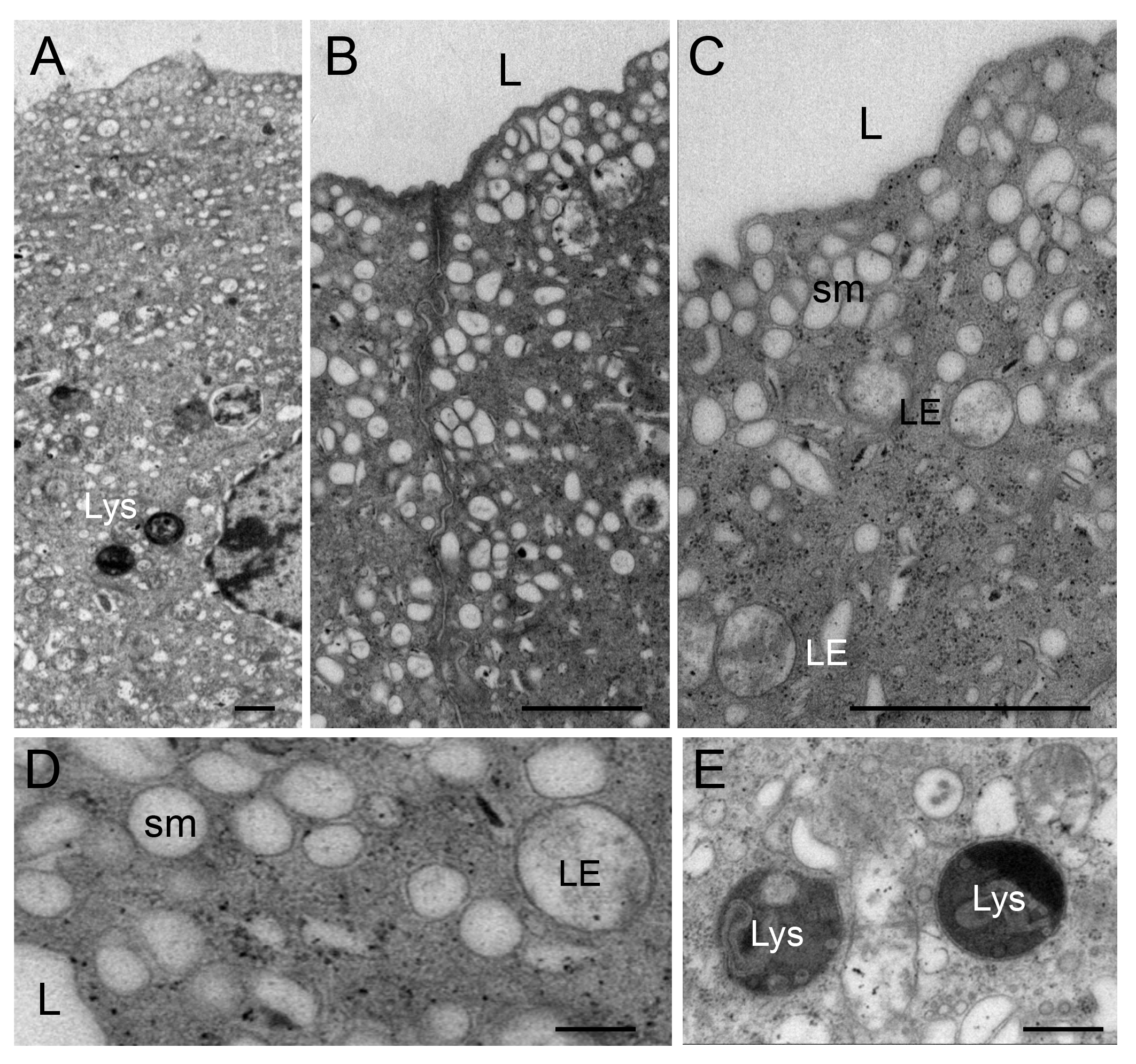

Supplement: Figure S2 — Uroplakin II-deficient mouse urothelium lacks the uroplakin-plaque lined multivesicular bodies. Note in (A to C) a lack of fusiform vesicles, apical uroplakin plaques, and multivesicular bodies, that are typical of normal urothelial umbrella cells (cf. Figs. 3A and 4A; [11], [12]). Rather, the cytoplasm of UPII-deficient superficial cells is filled with small MAL-positive vesicles (SV) that are involved in delivering the remaining UPIb/IIIa uroplakin pair to the apical surface [45]. Note in (D and E) that, despite the lack of MVBs, late endosomes (LE) and lysosomes (Lys) are clearly identifiable. Magnification bars = 1 µm in A-C, and 0.2 µm in D and E. (TIF) [file pone.0099644.s002.tif]
